# Chromosomal Characterization of the Three Subgenomes in the Polyploids of *Hordeum murinum* L.: New Insight into the Evolution of This Complex

**DOI:** 10.1371/journal.pone.0081385

**Published:** 2013-12-13

**Authors:** Ángeles Cuadrado, Alejandro Carmona, Nicolás Jouve

**Affiliations:** Departamento de Biomedicina y Biotecnología, Universidad de Alcalá, Alcalá de Henares, Madrid, Spain; The Centre for Research and Technology, Hellas, Greece

## Abstract

*Hordeum murinum* L. is a species complex composed of related taxa, including the subspecies *glaucum*, *murinum* and *leporinum*. However, the phylogenetic relationships between the different taxa and their cytotypes, and the origin of the polyploid forms, remain points of controversy. The present work reports a comparative karyotype analysis of seven accessions of the *H. murinum* complex representing all subspecies and cytotypes. The karyotypes were determined by examining the distribution of the repetitive *Triticeae* DNA sequences pTa71, pTa794, pSc119.2, pAs1 and pHch950, the simple sequence repeats (SSRs) (AG)_10_, (AAC)_5_, (AAG)_5_, (ACT)_5_, (ATC)_5_, and (CCCTAAA)_3_ via in situ hybridization. The chromosomes of the three subgenomes involved in the polyploids were identified. All tetraploids of all subspecies shared the same two subgenomes (thus suggesting them to in fact belong to the same taxon), the result of hybridization between two diploid ancestors. One of the subgenomes present in all tetraploids of all subspecies was found to be very similar (though not identical) to the chromosome complement of the diploid *glaucum*. The hexaploid form of *leporinum* came about through a cross between a tetraploid and a third diploid form. Exclusively bivalent associations among homologous chromosomes were observed when analyzing pollen mother cells of tetraploid taxa. In conclusion, the present results identify all the individual chromosomes within the *H. murinum* complex, reveal its genome structure and phylogeny, and explain the appearance of the different cytotypes. Three cryptic species are proposed according to ploidy level that may deserve full taxonomic recognition.

## Introduction

Polyploidy has been particularly important in the evolution of the family *Poaceae* [1]. Certainly, it has played an important role in the diversification of the genus *Hordeum*, which contains diploid, tetraploid and hexaploid taxa. C-banding karyotypes and the meiotic behaviour of *Hordeum* hybrids suggest the existence of four basic diploid genomes [2]–[4]: **H**, **I**, **Xa** and **Xu** (following the nomenclature of Wang et al. [5] and Linde-Laursen et al. [6]). Accordingly, molecular phylogenies cluster *Hordeum* species into four groups [7]. The species *H. murinum* L., which possesses the **Xu**-genome, is usually recognized as having three subspecies: *glaucum* (Steud.) Tzvelev (2n = 2x = 14), *murinum* (2n = 4x = 28), and *leporinum* (Link) Arcang. (2n = 4x = 28, 2n = 6x = 42) [8]. However, since there is no single diagnostic morphological characteristic that distinguishes the three forms - which are easily confused - most authors refer to the *murinum* complex [9], [10].

For many years it remained unclear whether the polyploid forms of *H. murinum* were auto- or allopolyploids [11]–[16]. Recently, however, allopolyploidy has been strongly supported by molecular phylogenetic analyses that differentiate 2x, 4x and 6x forms [17]–[19]. Indeed Jakob and Blattner [20] indicate that *glaucum* was involved in the formation of the tetraploids together with a now likely extinct taxon belonging to the same *Hordeum* **Xu** genome group, and that a third, closely related taxon contributed to the formation of the hexaploid *leporinum*. Nevertheless, additional cytogenetic analyses are needed to reveal the true genomic constitution of the different *H. murinum* taxa and cytotypes.

The aim of the present work was to examine the karyotypes of a representative sample of *H. murimum* accessions covering all subspecies and cytotypes. If the diploid *glaucum* was involved in the origin of the polyploid cytotypes, its chromosomes should be present in them. The same is true if tetraploid forms were involved in the origin of the hexaploid forms.

## Materials and Methods

Material representing all three subspecies and cytotypes of the *H. murinum* complex was obtained from the IPK Germplasm Bank (Gatersleben, Germany). Table 1 provides information on the accession numbers and places of origin of the material used.

**Table 1:** List of species studied.

*Hordeum murinum* L.			
Accession number	Scientific name	Country of origin	Chromosome number
BCC2002	*Hordeum murinum L. subsp. glaucum* (Steud.) Tzvelev	Tunisia	2n = 14
BCC2007	*Hordeum murinum L. subsp. leporinum* (Link) Arcang.	Spain	2n = 28
GRA1021	*Hordeum murinum L. subsp. leporinum* (Link) Arcang.	Italy	2n = 28
GRA1144	*Hordeum murinum L. subsp. leporinum* (Link) Arcang.	France	2n = 28
GRA1183	*Hordeum murinum L. subsp. murinum*	Armenia	2n = 42*
GRA2735	*Hordeum murinum L. subsp. glaucum* (Steud.) Tzvelev	Portugal	2n = 28*
GRA2894	*Hordeum murinum L. subsp. murinum*	Spain	2n = 28

* In disagreement with expected polyploidy level.

### Chromosome preparation

Root tips were obtained from seedlings and exceptionally from plants grown in pots in a greenhouse. Meiotic divisions were observed in pollen mother cells. Chromosome preparations were obtained as previously described [21].

### Probes, labeling and in situ hybridization

Five probes containing repeated DNA sequences were used to characterize chromosomes by FISH: pTa794 and pTa71 (respectively containing 5S rDNA and 45S rDNA from *Triticum aestivum* L.), pSc119.2 and pAs1 (tandem repeat sequences obtained from *Secale cereale* L. and *Aegilops tauschii* Coss respectively) and pHch950 (a dispersive, repetitive sequence derived from *Hordeum chilense* Roem. & Schult.). Full probe descriptions, probe labelling procedures and the FISH conditions used have been described in earlier work [15]. To detect telomeric repeats (Tel.), the oligomer (5′-CCCTAAA-3′)_3_, synthesized with Dy547 (red) (Isogen Life Science) at both ends, was used according to Cuadrado et al. [22]. Five other synthetic oligonucleotides - (AG)_10_, (AAC)_5_, (AAG)_5_, (ACT)_5_ and (ATC)_5_ - synthesized with biotin (Roche Applied Science) at both ends - were used to detect their respective SSRs by ND (non denaturing)-FISH, as previously described [23].

### Fluorescence microscopy and imaging

Slides were examined using a Zeiss Axiophot epifluorescence microscope. Biotin/Cy3, digoxigenin/FITC and DAPI stained images were recorded with each filter set using a cooled CCD camera (Nikon DS). The localization of the signals relative to the DAPI staining pattern was resolved by merging images using Adobe Photoshop, employing only those functions that applied equally to all pixels.

## Results

### Ploidy level of *H. murinum* taxa

In agreement with previous chromosome counts for *H. murinum* taxa, diploid (2n = 2x = 14), tetraploid (2n = 4x = 28) and hexaploid (2n = 6x = 42) cytotypes were detected among the examined accessions (Table 1). However, accession GRA2735 - supposedly *glaucum* - was found to have 2n = 28 instead of the expected 2n = 14. Further, accession GRA1183 – supposedly *murinum* – was found to have 2n = 42 instead of the expected 2n = 28. Individuals of these accessions were therefore grown in the field and identified morphologically following the key provided by Bothmer et al. [8]. Accession GRA2735 showed features diagnostic of *glaucum*, including pedicelate central spikelets longer than the lateral spikelets, and anthers with purple dots (Fig. S1). This is the first report of a tetraploid form of *glaucum*. Accession GRA1183, however, showed the diagnostic characteristics of *leporinum* (pedicelate central spikelets shorter than the lateral spikelets).

### Karyotype analysis of diploid *H. murinum*

**Figure 1:**
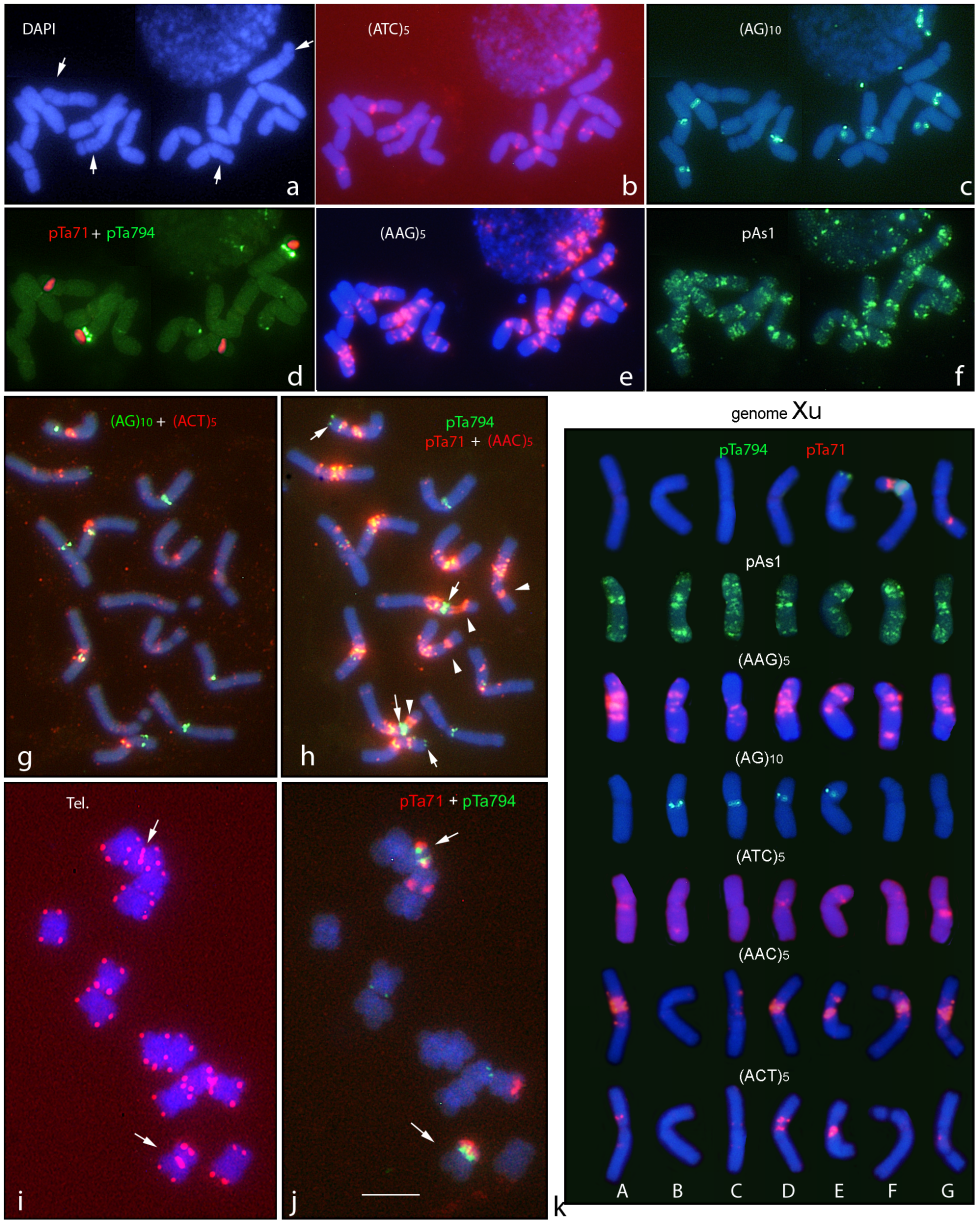
*In situ* hybridization with probes pTa71 (45S rDNA), pTa794 (5S rDNA), pAs1, (AG)_10_, (AAC)_5_, (AAG)_5_, (ACT)_5_, (ATC)_5_, and the telomeric (Tel.) probe (CCCTAAA)_3_, in three metaphases (panels a–f, g–h and i–j respectively) of the diploid *H. murinum* accession BCC2002. Each panel shows merged images to facilitate the visualization of the *in situ* signals with respect to DAPI (blue) staining (**a**). In **a**, the arrows point to the two pairs of satellited chromosomes; in **h**, the arrows and arrowheads point to 5S rDNA and 45S rDNA loci respectively. In **i** and **j**, the arrows point to the pair of chromosomes carrying interstitial telomeric repeats and both ribosomal sequences. (**k**) Karyotypes showing one chromosome of each homologous group chosen from the metaphases shown in **g–h** (top and two bottom rows) and **a–f** (the remaining rows). Scale bar = 10 µm.

Figure 1 shows the distinctive hybridization patterns obtained with a number of repetitive probes in metaphase chromosomes of the *H. murinum* diploid accession BCC2002.

FISH analysis using probe pTa71 revealed four signals at the secondary constriction of the two satellited chromosome pairs (Fig. 1a). One of these chromosome pairs also carried a pTa794 signal in a more proximal position (Fig. 1d). Another pair of chromosomes carrying a pTa794 signal in a distal position on the short arm was detected (Fig. 1d and j). Thus, the combination of pTa71 and pTa794 easily distinguishes three chromosome pairs (Fig. 1k, top row). The pAs1 probe returned similar banding patterns for different chromosomes, making clear identifications very difficult (Fig. 1f). No signals were observed with probe pSc119.2.

The most clear, intense and rich pattern of signals was obtained with probe (AAG)_5_, which clearly identified the seven homologues chromosome pairs (Fig. 1e). (AAC)_5_ produced similar though slightly less diagnostic patterns (Fig. 1h). Most of the AAC sites co-localized with AAG in the pericentromeric regions. Well defined and intense signals were observed with (AG)_10_ near the centromeres of four chromosome pairs (Fig. 1c). In contrast (ATC)_5_ (Fig. 1b) and (ACT)_5_ (Fig. 1g) only revealed signals of weak intensity that were little suitable as diagnostic markers. Finally, the expected signals for the telomeric probe were seen at the ends of all chromosome arms. In addition, interstitial telomeric repeats were found in the satellited chromosome pair carrying both pTa71 and pTa794 signals (Fig. 1i–j).

Once the hybridisation pattern of each probe was analysed, multiple target in situ experiments using two-by-two combinations of the probes (reprobing the same chromosome preparations) allowed the characterization of the seven chromosome pairs (e.g., Fig. 1a–f). Following the classical nomenclature used for *Triticeae* chromosomes with unknown homoeology, karyotypes were constructed arranging chromosomes A to G in order of decreasing length with the satellited chromosomes at the end (Fig. 1k).

### Chromosome identification and karyotype diversity within tetraploid *H. murinum*

**Figure 2:**
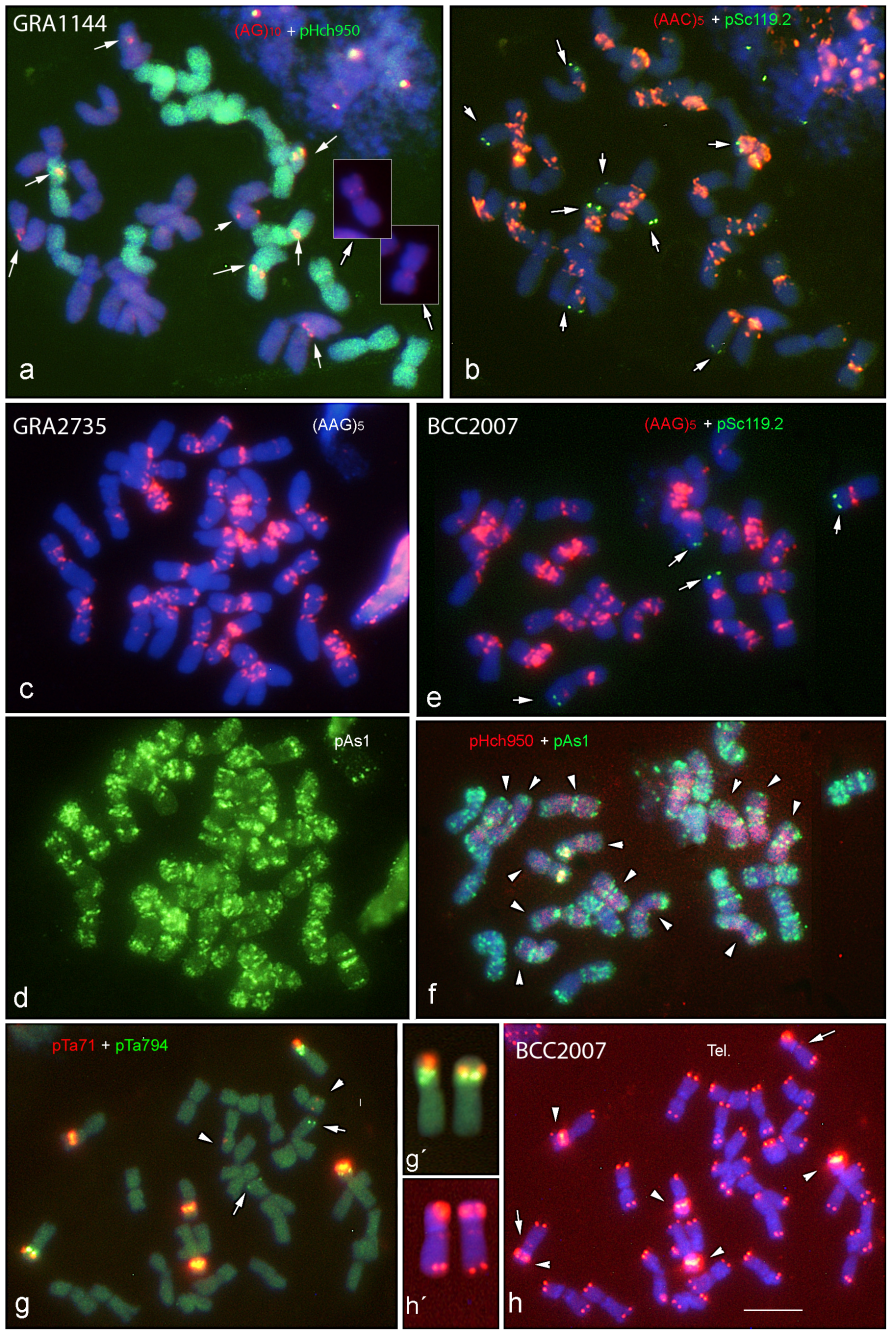
*In situ* hybridization in accessions GRA1144 (a–b), GRA2735 (c–d) and BCC2007 (e–h) illustrating the patterns obtained with probes pHch950, pTa71, pTa794, pSc119.2, pAs1, (AAG)_5_, (AAC)_5_, (AG)_10_ and the telomeric (Tel.) probe (CCCTAAAG)_3_ in tetraploid *H. murinum* taxa. In **a**, the arrows point to (AG)_10_ signals in five pairs of chromosomes. One pair carrying weak signals is shown in the insets. In **b** and **e**, the arrows point to pSc119.2 signals. The arrowheads in **f** point to the 14 chromosomes labeled with pHch950. In **g**, the arrows and arrowheads point to minor pTa794 and pTa71 sites respectively. In **h**, the arrows and arrowheads point to interstitial telomeric repeats and stronger, derived pTa71 signals (see **g**) respectively. The satellited chromosome pair belonging to subgenome **Xu** carrying pTa71, pTa794 and interstitial telomeric sequences is amplified in **g′** and **h′**. Scale bar = 10 µm.

Figures 2 shows the distinctive hybridization patterns obtained with several repetitive probes in metaphase chromosome preparations of five tetraploid accessions covering all *H. murinum* subspecies.

In all tetraploid accessions, probe pHch950 hybridized in a dispersed fashion with seven chromosome pairs (Fig. 2a and f). Six intense pTa71 signals were detected at the secondary constriction of the three satellited chromosome pairs. Two further minor pTa71 signals were seen on the long arms of a small metacentric chromosome pair (Fig. 2g). Six pTa794 signals were detected, the four strongest in two satellited chromosome pairs (Fig. 2g). Besides those expected at both chromosome ends, telomeric interstitial signals were observed on one of the two satellited chromosome pairs carrying (as in diploids) pTa71 and pTa794 signals (Fig. 2g–h).

pSc119.2 revealed subtelomeric signals of different intensity ranging in number from four on two chromosome pairs in accessions GRA1021 and BCC2007, to ten on four chromosome pairs in accession GRA2894 (compare Fig. 2b and e). This indicates diversity among the 4x accessions for the presence/absence of this repetitive sequence. Similarly, (AG)_10_ showed well defined signals of different intensity in different chromosomal regions (from the centromere to a subtelomeric position) in five or six chromosome pairs, depending on the accession analyzed (Fig. 2a).

**Figure 3:**
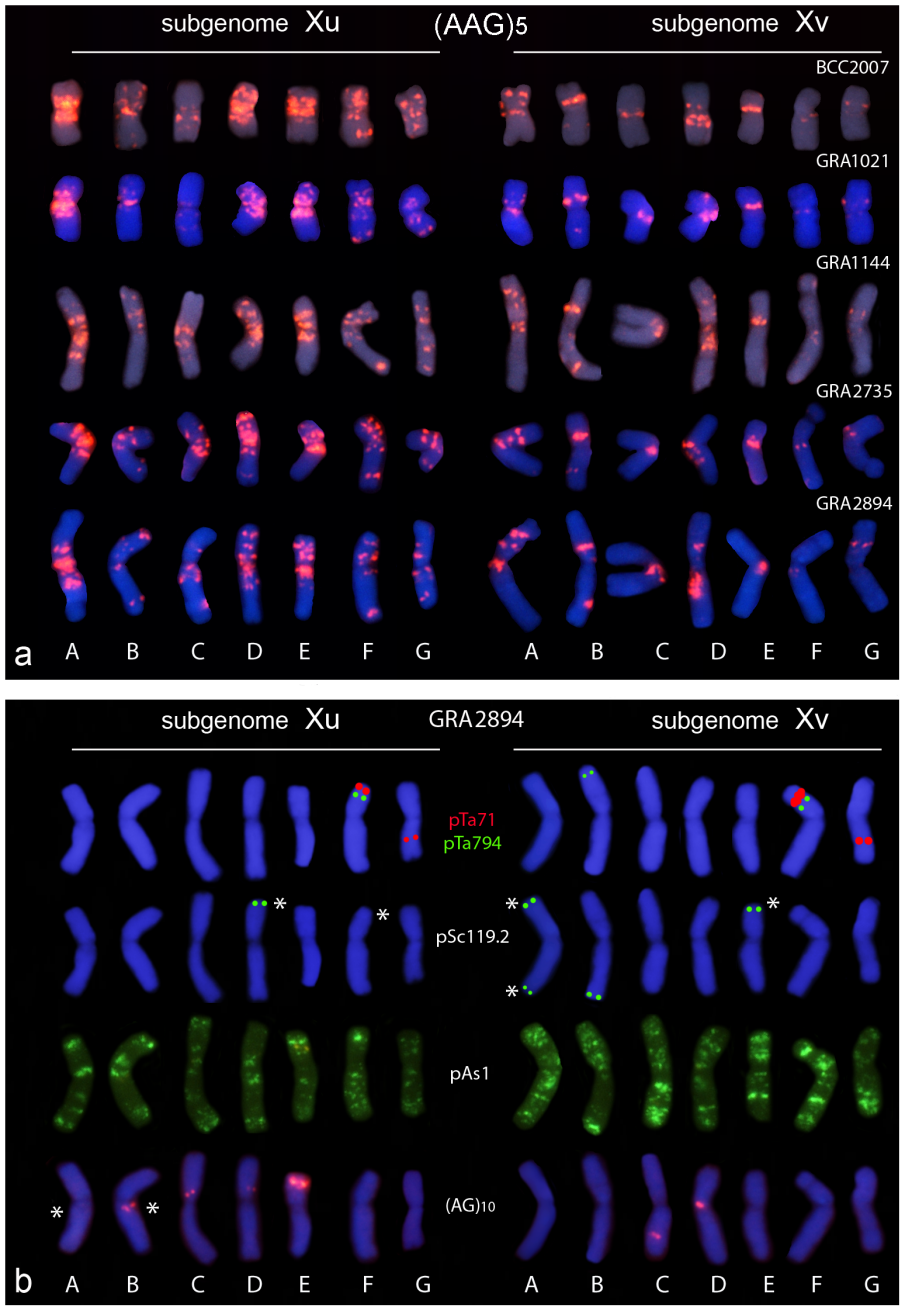
(a) (AAG)_5_ karyotypes from each tetraploid genotype studied showing one chromosome of the seven homologous groups in the Xu and Xv subgenomes. Chromosomes of each karyotype were chosen from the same metaphase. Fig. 2c and e show those of GRA2735 and BCC2007. Note that the *in situ* patterns for each accession - even those belonging to different *H. murinum* subspecies - are very similar to one another. (b) Karyotypes of accession GRA2894, providing a representative sample of the chromosomal distribution of different probes in tetraploids of *H. murinum*. Note that signals from probes pTa71, pTa794 and pSc119.2 were drawn over the DAPI-stained chromosomes. Asterisks indicate the polymorphic sites (presence/absence) observed among the tetraploid accessions (Table 2).

The pAs1 probe revealed a rich pattern of signals on all chromosome arms, making individual chromosome identification very difficult (Fig. 2d and f). However, all 14 chromosomes pairs were easily identified on the basis of the chromosomal location of (AAG)_5_ (Fig. 2c–e and Fig. 3a). The (AAC)_5_ probe mostly co-localized with (AAG)_5_ but produced a less diagnostic pattern (Fig. 2b). Finally, as in the diploids, no reliable (ATC)_5_ or (ACT)_5_ signals were observed; no attempt was therefore made to characterize their *in situ* patterns in detail.

The results obtained with the pHch950 probe were particularly important for identifying the chromosomes of the two subgenomes involved in the tetraploids (Fig. 2a and f). Firstly, the seven chromosome pairs revealed with the pHch950 probe were analyzed in detail with the diagnostic pTa71, pTa794 and (AAG)_5_ probes. With the exception of the non-satellited chromosome pair carrying the minor 45S rDNA locus, the chromosomes showed sizes, morphologies and hybridization patterns similar to those seen for the chromosomes of diploid *H. murinum* (compare Fig. 1k and Fig. 3a). The above seven pairs were identified as belonging to subgenome **Xu** and were arranged in the karyotypes according to the nomenclature system previously employed for the diploids. The pair with no counterpart in the diploid was identified as G Although other minor differences were found between the **Xu** subgenome and the genome of the diploids (for example, FISH with the probe pTa794 returned no signal on chromosome E of the tetraploids), the physical map returned by the probes revealed great similarities between the diploid *glaucum* and the subgenome **Xu** of the tetraploids (compare Fig. 1k and Fig. 3b). For example, the satellited chromosome pair of this subgenome carries both 45S rDNA and 5S rDNA loci and interstitial telomeric sequences, just like the satellited chromosome F of diploids (compare Figs. 1j and 2g–h).

Once the identity of the **Xu** chromosomes was established, the seven chromosome pairs without pHch950 signals were characterized. These chromosomes should not share the same **Xu** genome, and the provisional designation of subgenome **Xv** is here proposed. Among the **Xu** and **Xv** chromosomes, homoeology relationships could be established for the two pairs of satellited chromosomes: chromosomes F (which, like chromosome F of diploids and subgenome **Xu** of tetraploids, carries both rDNA sequences) and G (which, like chromosome G of the diploids has a large satellite on the long arm) (Fig. 2g). The remaining chromosomes of subgenome **Xv** (A to E) were arranged in decreasing order of the length; however, no homoeologies can be established between these chromosomes and their counterpart in the subgenome **Xu** without further genetics analysis.

Finally, the easy identification of all 14 chromosome pairs after ND-FISH with (AAG)_5_ allowed chromosome-by-chromosome analysis of the hybridization patterns of the remaining probes (Fig. 2 e–f), and the assessment of cytogenetic diversity by comparing the homologous chromosomes of the five tetraploid accessions (Fig. 3a). Accession GRA2894, which showed the largest number of pSc119.2 and (AG)_10_ signals among the tetraploids, was chosen to represent the karyotypes based on probes pTa71, pTa794, pAs1, pSc119.2 and (AG)_10_ (Fig. 3b). The results are summarized in Table 2.

**Table 2:** Physical localization of four probes used for cytological characterization of *H. murinum* chromosomes.

Accession					Probes											
*Subspecies*	pTa71				pTa794				pSc119.2				(AG)_10_			
	**Xu**				**Xu**				**Xu**				**Xu**			
**BCC2002**	FS				ES								BL			
*glaucum*	GL				FS								CS			
													DS			
													ES			
	**Xu**		**Xv**		**Xu**		**Xv**		**Xu**		**Xv**		**Xu**		**Xv**	
**BCC2007**	FS		FS		FS		BS				BL		CS		CL	
*leporinum*	GL		GL				FS				ES		DS		DS	
													ES			
**GRA1021**	FS		FS		FS		BS		FS		BL		CS		CL	
*leporinum*	GL		GL				FS						DS		DS	
													ES			
**GRA1144**	FS		FS		FS		BS		FS		AL		CS		CL	
*leporinum*	GL		GL				FS				BLES		DSES		DS	
**GRA2735**	FS		FS		FS		BS				AL		AL		CL	
*glaucum*	GL		GL				FS				BL		CS		DS	
											ES		DS			
													ES			
**GRA2894**	FS		FS		FS		BS		DS		AS		BL		CL	
*murinum*	GL		GL				FS				AL		CS		DS	
											BL		DS			
											ES		ES			
	**Xu**	**Xv**		**Xw**	**Xu**	**Xv**		**Xw**	**Xu**	**Xv**		**Xw**	**Xu**	**Xv**		**Xw**
**GRA1183**	FS	FS		FS	FS	BS		FS	FL	AS			BL	CL		AS
*leporinum*	GL	GL		GL		FS				AL			CS	DS		BL
										ES			DS			CL
										GS			ES			DS
																EL
																FS

Genomes (**Xu**, **Xv** and **Xw**); Chromosomes (A to G); Arms (S = short; L = long).

### Identification of the three subgenomes presents in hexaploid *H. murinum*

**Figure 4:**
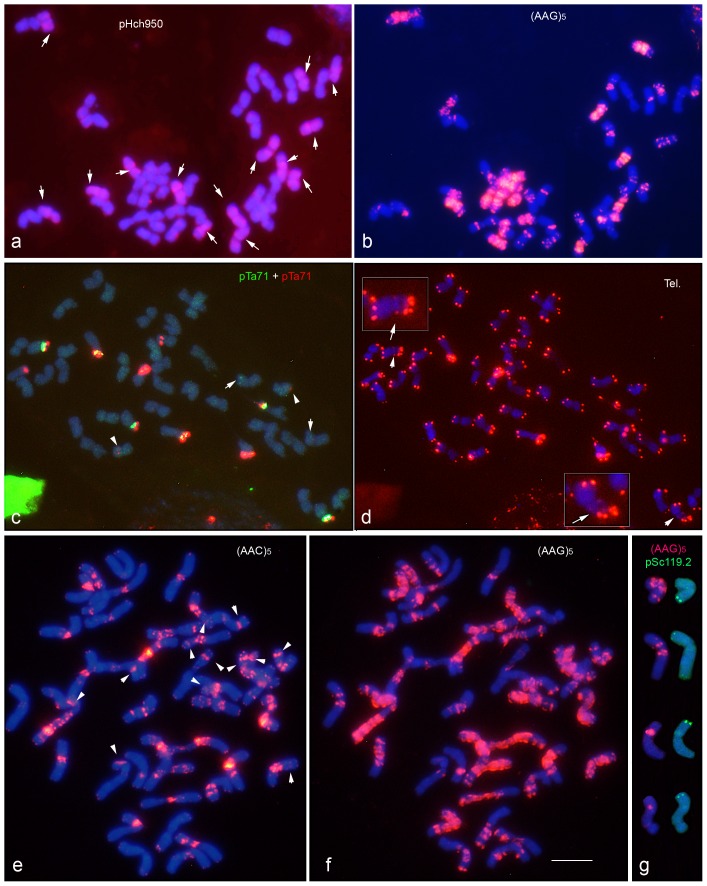
*In situ* hybridization with probes pHch950, pTa71 (45S rDNA), pTa794 (5S rDNA), pSc119.2, (AAG)_5_, (AAC)_5_, and the telomeric (Tel.) probe (CCCTAAA)_3_, in three metaphases cells (panels a–b, c–d and e–g, respectively) of the hexaploid *H. murinum* accession GRA1183. Each panel shows merged images to facilitate the visualization of the signals with respect to the blue DAPI staining. In **a**, the arrows point to the 14 chromosomes revealed with pHch950. In **c**, the arrows and arrowheads point to the 5S rDNA and 45S rDNA minor loci respectively. In **d**, the arrows point to interstitial telomeric repeats (enlarged in the insets). In **e**, the arrowheads indicate pTa71 signals derived from previous hybridizations. (**g**) The four chromosome pairs carrying pSc119.2 signals were chosen from the metaphase shown in **e–f**. Scale bar = 10 µm.

**Figure 5:**
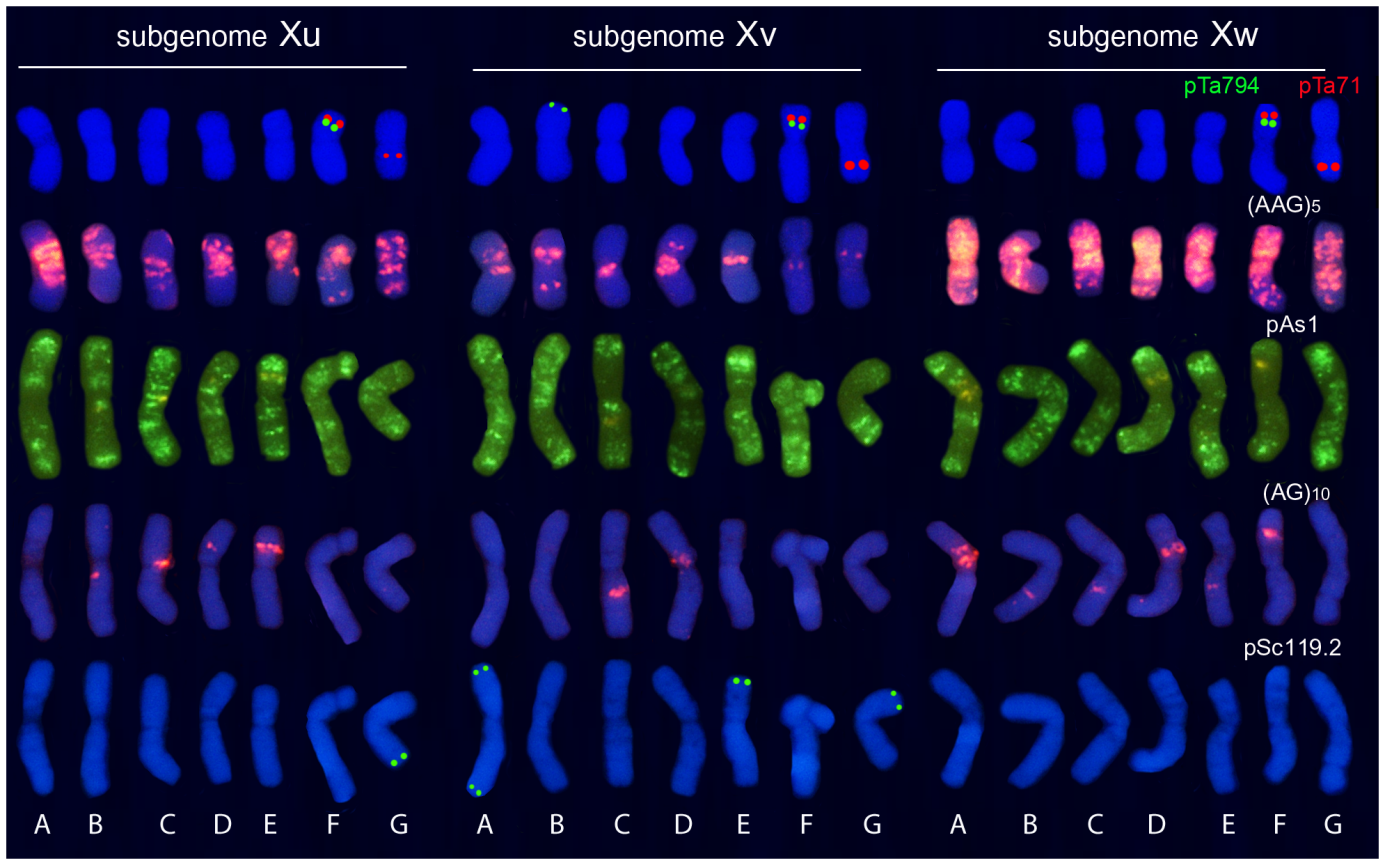
Karyotypes of hexaploid *H. murinum* accession GRA1183 probed with pTa71, pTa794, (AAG)_5_, pAs1, (AG)_10_ and pSc119.2, showing one chromosome of the seven homologous groups of the Xu, Xv and Xw subgenomes. Chromosomes of each karyotype were chosen from the same metaphase (that shown for [AAG]_5_ in Fig. 4b). Note that the pTa71, pTa794 and pSc119.2 signals were drawn over the DAPI-stained chromosomes. The orange signals in the pAs1 karyotype are the stronger red signals observed in the (AG)_10_ karyotype.

Figure 4 shows the distinctive hybridization patterns obtained with seven probes in metaphase chromosomes of the hexaploid accession GRA1183. Karyotypes for six probes were constructed (Fig. 5).

As in the tetraploids, seven chromosomes pairs were distinguished using pHch950 (Fig. 4a). The pTa71 probe revealed 10 strong signals at the secondary constriction of the five satellited chromosome pairs. Two minor pTa71 signals were also seen on a small metacentric chromosome pair (Fig. 4c). Eight pTa794 signals were detected, the six strongest in three satellited chromosomes pairs (Fig. 4c). As in the diploid and tetraploid taxa, one satellited chromosome pair (carrying both ribosomal loci) showed interstitial telomeric signals (Fig. 4d). The chromosomal distribution revealed by pAs1 was coincident with that seen for the diploids and tetraploids, although in the hexaploids (with more chromosomes of similar morphology and *in situ* patterns) it was very hard to identify individual chromosomes (Fig. 5). Ten pSc119.2 signals of different intensity were localized in subtelomeric regions of four chromosome pairs (Fig. 4g).

Once again, among the SSR probes investigated, the richest pattern of signals was obtained with probe (AAG)_5_ (Fig. 4b and f). (AAC)_5_ signals mostly co-localized with clusters of AAG repeats, although some chromosome were very enriched in (AAG)_5_ signals and showed no accompanying (AAC)_5_ signal (compare Fig. 4e–f). As in the diploid and tetraploid accessions, no very reliable signals were observed with (ACT)_5_ and (ATC)_5_. Finally, discrete (AG)_10_ signals were observed in different locations in 12 chromosome pairs (Fig. 5).

The same strategy used for the characterization of the subgenomes in the tetraploids was followed in the hexaploids. Firstly, the seven chromosome pairs revealed with pHch950 were analyzed in detail with the diagnostic probes pTa71, pTa794 and (AAG)_5_. This set of chromosomes has the same morphology and *in situ* patterns as the chromosomes of the **Xu** subgenome of the tetraploids. The 14 chromosome pairs that showed no hybridization with probe pHch950 were easily separated into two groups of seven chromosome pairs. One group showed the same morphology and *in situ* patterns as the chromosomes of the subgenome **Xv** observed in the tetraploids, and was easily identified (Fig. 4b). The other group, whose members were very enriched in (AAG)_5_ signals, belongs to a related genome here named subgenome **Xw** (Fig. 5). Probes pTa794 and pTa71 diagnostically identified two chromosomes belonging to subgenome **Xw**. Like F chromosomes belonging to subgenomes **Xu** and **Xv**, the pair of satellited chromosomes carrying proximal 5S rDNA sites was identified as chromosome pair F. The other pair of **Xw** satellited chromosomes carrying only 45S rDNA was identified as chromosome pair G (Fig. 4c). The remaining chromosomes belonging to the **Xw** subgenome (A to E) were arranged in the karyotype on the basis of chromosome size. Once again, no homoeologies could be established between these chromosomes and the respective **Xu** and **Xv** chromosomes.

Finally, the identification of chromosomes with (AG)_10_ and pSc119.2 signals was performed after reprobing with (AAG)_5_ (Fig. 4g). (AG)_10_ was found on chromosomes belonging to all three subgenomes (the positions on **Xu** and **Xv** were the same as seen on the chromosomes of the tetraploids). It is noticeable that, as in the diploids (genome **Xu**), no signals were observed with the probe pSc119.2 on chromosomes belonging to subgenome **Xw** (Fig. 5, Table 2)

### Meiotic behaviour of chromosomes

**Figure 6:**
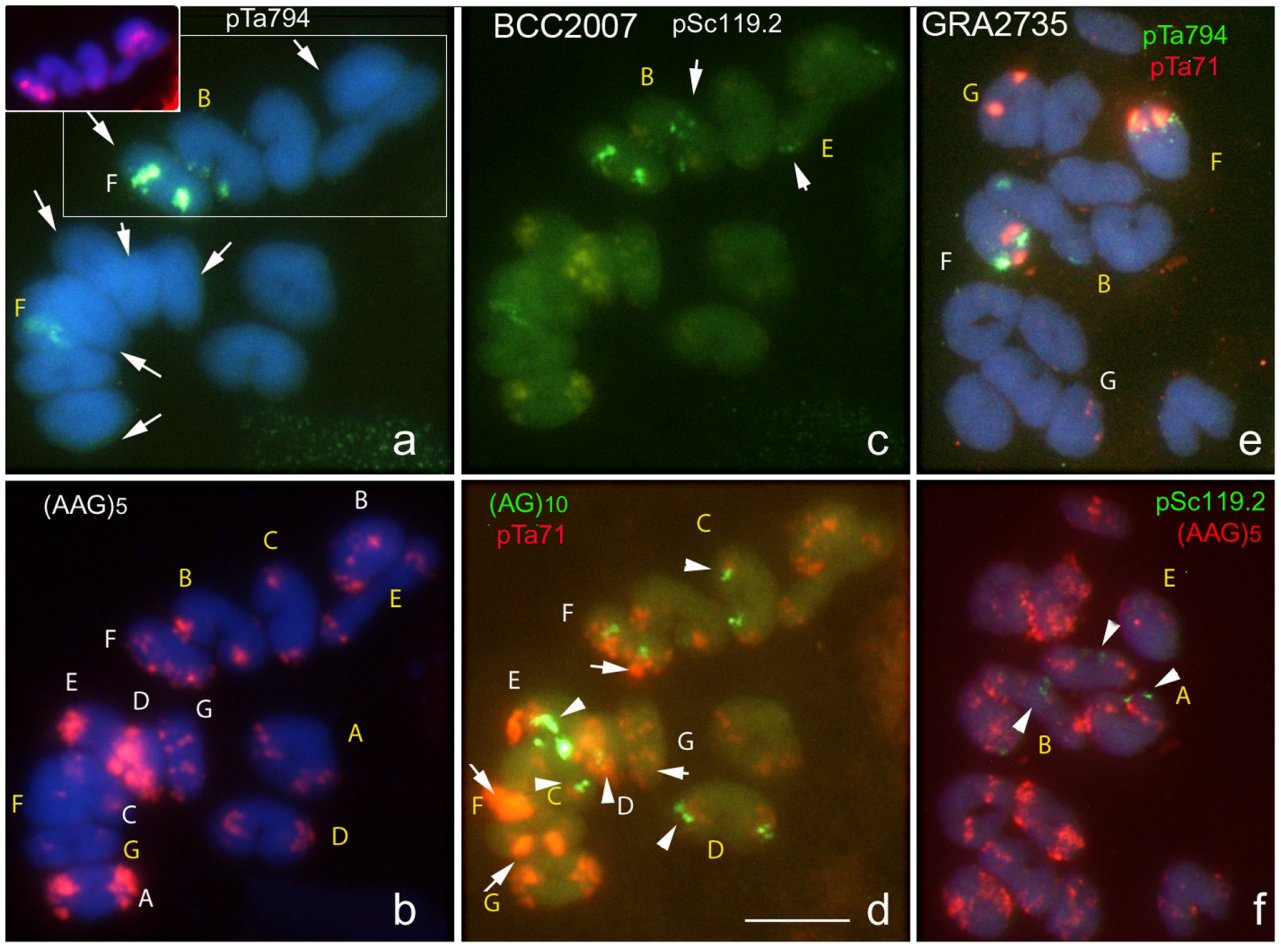
Photomicrographs showing meiotic metaphase I chromosome spreads of pollen mother cells from the tetraploid accessions BCC2007 (a–d) and GRA2735 (e–f) probed with multiple repeat DNA in sequential hybridizations. The chromosome identities present as bivalents Xu-Xu and Xv-Xv are indicated in white and yellow letters, respectively. In **a**, the arrows point the seven bivalents revealed with pHch950 (some of them in the inset). In **c**, the arrows point the bivalents carrying pSc119.2 signals. In **d**, the arrows and arrowheads point to the (AG)_10_ and 45S rDNA signals respectively. In **f**, the arrowheads point to the pSc119.2 signals. Scale bar = 10 µm.

The meiotic behaviour of the chromosomes was analyzed in pollen mother cells at metaphase I in tetraploids BCC2007 and GRA2735. Only bivalents were formed (mostly ring-type); no univalents, multivalents or other chromosome associations were observed. Sequential experiments using two-by-two combinations of the probes unequivocally identified the seven bivalents. In both accessions, the bivalents only involved homologous chromosomes (Xu-Xu and Xv-Xv) (Fig. 6).

## Discussion

### Identification of *H. murinum* chromosomes

The first step in understanding the genome structure and evolution of a species is the unambiguous discrimination of its chromosomes. The identification of each *H. murinum* chromosome pair, including its hexaploids, is here reported for the first time. As indicated by other authors, the five *Triticeae* probes used (pTa71, pTa794, pAs1, pHch959 and pSc119.2) were insufficient for the reliable identification of most chromosomes [15], [18], [24]. However, the pattern of distribution of several SSRs was shown very useful in chromosome identification. Indeed, the use of (AAG)_5_ in combination with the morphology of DAPI-stained chromosomes was enough to easily distinguish all individual chromosomes. The use of a single probe for chromosome identification facilitates the co-localization of other probes carrying different fluorochromes in two-colour *in situ* experiments. With the exception of (ACT)_5_ and (ATC)_5_ (which were ineffective as chromosome markers), the probes used in the present work provided a saturated physical map of *H. murinun* with a rich set of cytogenetic landmarks distributed throughout all chromosome arms.

It is well documented that several members of the tribe *Triticeae* commonly show 5S rDNA loci in homoeologous group 5 (e.g., wheat, rye and *H. bulbosum*), while 45S rDNA loci are commonly present in homoeologous groups 5 and 6 (e.g., barley and *H. chilense*) [25]–[27]. On the basis of the location of rDNA loci, chromosomes F and G of (sub)genomes **Xu**, **Xv** and **Xw** should be assigned respectively to the *Triticeae* homoeologous groups 5 and 6. However, homoeologies can only be fully supported by demonstrating conserved synteny in further analysis. Since *H. murinum* chromosomes can be identified when in bivalent configuration (Fig. 6), the analysis of meiotic pairing in the hybrids of *H. murinum* with *H. vulgare* should allow homoeologous relationships to be established between the species, as reported for *H. bulbosum* when analyzing its hybrids with *H. vulgare* [27].

### The origin of polyploids in the *H. murinum* complex

The confluence of distinguishable groups of seven chromosome pairs in the tetraploids and hexaploids contributes to our knowledge on the origin of the polyploid forms of *H. murinum*. The present results show the morphology and *in situ* patterns of the chromosomes in the diploid *glaucum* to be very similar to a set of 14 chromosomes in the tetraploid and hexaploid cytotypes. This supports the idea that *glaucum* was the diploid donor of the subgenome **Xu** present in *H. murinum* polyploids, and agrees with the assumption of Rajhathy and Morrison [11] (based on studies of meiosis) that one of the genomes of the *murinum* tetraploid cytotypes is that of diploid *glaucum*.

Nearly nothing is known about the identity of the non-*glaucum* diploid progenitors. Recent molecular phylogenies have shown that ancient *H. murinum* diploid progenitors involved in the origin of polyploids should belong, along with *glaucum*, to the same **X**u genome group of *Hordeum* species that became isolated after the separation of their sister group, i.e., the **H** genome lineage of *H. vulgare* and *H. bulbosum* [17], [19], [28]. The present results seem to reflect the existence of substantial differences in the amount and distribution of certain repeat DNA sequences among the subgenomes present in polyploids. Thus, even assuming that the **Xu**, **Xv** and **Xw** genomes have a monophyletic origin, their separation from a common ancestor must have occurred at a relatively early stage. A detailed chromosome analysis of other diploid forms of *Hordeum* and closely related *Triticeae* species might provide new clues regarding the identity of the **Xv** and **Xw** donor progenitors, or support the hypothesis suggested by Jacob and Blattner [20] that these genomes belong to extinct species. Irrespective of the identity of the non-*glaucum* parents, the donor species of the subgenome **Xv** must have been common to all tetraploids of all subspecies. Indeed, only minor differences were seen when comparing karyotypes for the analyzed probes of the five tetraploid accessions examined. The *in situ* patterns of the **Xu** and **Xv** subgenome chromosomes of the hexaploids are almost identical to the corresponding chromosomes present in tetraploids, indicating a common origin and low divergence after the addition of the third **Xw** subgenome at the hexaploid level.

### Genome remodelling and polyploidy

Allopolyploidy has been an important mechanism in the rapid genomic evolution of the members of *Triticeae* [29]–[31]. The present work contributes to our understanding of the coevolution of the **Xu**, **Xv** and **Xw** subgenomes following the polyploidization process. In general, the chromosomes of the same (sub)genome at different ploidy levels are very similar, suggesting that no great karyotypic alteration occurred after polyploidization. Only chromosome G of subgenome **Xu** present in the polyploids appears to have no definitive counterpart in diploid *glaucum*. This agrees with the fact that diploid *glaucum* has two pairs of satellited chromosomes (F and G), as revealed by the presence of secondary constrictions and major 45S rDNA signals (Fig. 1k, [24]), while only one such pair (chromosome F) is present in the **Xu** subgenome of the polyploids [15], [18]. Genome remodelling after polyploidization could have led to the replacement of the satellited chromosome G observed in the diploids (with major 45S rDNA sites) by the small metacentric chromosome with only minor 45SrDNA signals found in the tetraploid and hexaploid cytotypes. Deletion of the 45S rDNA genes is likely the main cause of this karyotypic difference. In fact, deletion or inactivations of ribosomal genes are common in hybrid and polyploid members of *Triticeae*. Intergenomic translocations are also common structural changes detected in allopolyploids. An example of this is the well characterized cyclic translocation of chromosomes 4A, 5A and 7B in tetraploid and hexaploid wheats [32]. No large translocations were detected in the present study. However unlike diploid *glaucum*, which shows no pSc119.2 signals [24], [33], pSc119.2 signals were observed in subtelomeric positions on one **Xu** chromosome arm of three tetraploids and the hexaploid accessions analyzed in the present work. This suggests the presence of a terminal translocation or perhaps simply the jumping of transposable elements after allopolyploidization. The presence of the pSc119.2 signals in the same location on chromosome F**Xu** as seen in two *leporinum* accessions (from Italy and France) might indicate a common origin for these samples. In contrast, the interstitial telomeric repeats on chromosome F**Xu**, invariably found in the seven accessions here analyzed, suggest that this chromosomal rearrangement must be an ancient characteristic of the **Xu** genome. This may have occurred before the diversification of the *H. murinum* species but after the diversification of genomes **Xu** and **H** since no interstitial telomeric repeats are seen in barley [22].

### Taxonomy of the *H. murinum* complex

The *H. murinum* complex is probably one of the best studied groups in the genus *Hordeum*. However, the taxonomic treatment of *H. murinum* taxa has always been controversial. Although it is well accepted that *H. murinum* can be split into three subspecies - *glaucum*, *murinum* and *leporinum* [8] – their geographical and ecological distinctiveness has led some authors to contemplate three separate species: *H. glaucum* Steudel, *H. murinum sensu stricto* and *H. leporinum* Link [34], [35]. Another problem of the complex is the taxonomic status of the different cytological forms of *leporinum* (which are not recognized as distinct taxa since they show no distinctive morphological traits). An unexpected result of the present work was the finding of 28 chromosomes in accession GRA2735, classified as *glaucum* by the seed bank delivery information and confirmed as such by the information in Fig. S1. This is the first report of a tetraploid form of *glaucum*, which further reflects the difficulties encountered in dealing with the complex.

The present cytogenetic results showed no greater similarity to exist between the three *leporinum* tetraploid accessions than between tetraploids of the different subspecies. This agrees with many phylogenetic studies that have been unable to separate tetraploids of *leporinum* and *murinum*. Indeed, populational analyses of several quantitative and qualitative features, and indeed of different molecular markers, have revealed no significant differentiation between tetraploid populations morphologically classified as different taxa [9], [35]–[37]. Some authors suggest that the different subspecies form a continuous morphological cline strongly correlated with bioclimatic variables [18]. Others suggest the existence of hybrids of *murinum* and *leporinum* [37], while yet others indicate the separation of these taxa must have occurred relatively recently [20]. The present results suggest that all *murinum* tetraploids should be included within the same species.

In conclusion, this study reveals the origin of the polyploids of the *H. murinum* subspecies and highlights their phylogenetic relationships. It is here suggested that the members of the complex be divided into three subgroups according to their ploidy level: *2x-murinum*, *4x-murinum* and *6x-murinum*. *2x-murinum* with the haploid genome formula **Xu**, should include the diploid form of *glaucum*; *4x-murinum*, with the haploid genome formula **XuXv**, should include the currently recognized *murinum* and the tetraploid cytotypes of *glaucum* and *leporinum*. Finally, *6x-murinum* with the haploid genome formula **XuXvXw**, should include the hexaploid cytotypes of *leporinum*. Certainly, the taxonomic treatment of *H. murinum* based exclusively on morphological criteria would appear to be questionable.

## Supporting Information

Figure S1 Spikelets and anthers from the tetraploid accessions GRA2735 as distinctive features of *glaucum*. **a**) Pedicelate central spikelet longer than its lateral counterparts. **b**) The three anthers from a central spikelet showing characteristic purple spots.
(TIF)
