# Supplementary figures and images for: Chromosomal Characterization of the Three Subgenomes in the Polyploids of Hordeum murinum L.: New Insight into the Evolution of This Complex
Source: PLoS One. 2013 Dec 13;8(12):e81385. doi: 10.1371/journal.pone.0081385 (PMC3862567; doi:10.1371/journal.pone.0081385)

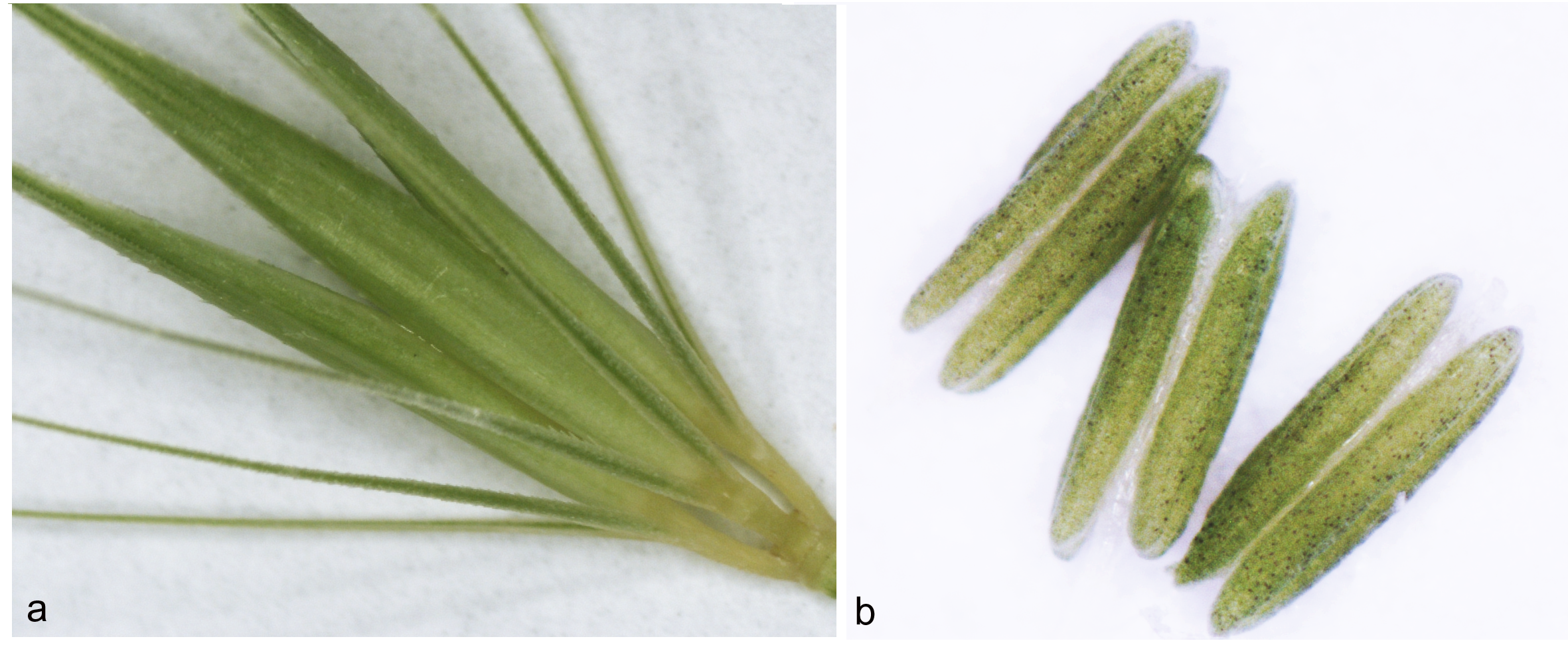

Supplement: Figure S1 — Spikelets and anthers from the tetraploid accessions GRA2735 as distinctive features of glaucum. a) Pedicelate central spikelet longer than its lateral counterparts. b) The three anthers from a central spikelet showing characteristic purple spots. (TIF) [file pone.0081385.s001.tif]
